# Malaria community case management usage and quality of malaria care in a moderate *Plasmodium falciparum* burden region of Chadiza District, Zambia

**DOI:** 10.1186/s12936-024-05047-1

**Published:** 2024-08-01

**Authors:** Erika Wallender, Bupe Kabamba, Marie-Reine I. Rutagwera, Chabu Kangale, John M. Miller, Travis Porter, Maximillian Musunse, Sarah Gallalee, Adam Bennett, Paul Psychas, Julie R. Gutman, Busiku Hamainza, Julie Thwing

**Affiliations:** 1 Epidemic Intelligence Service, U.S. Centers for Disease Control and Prevention, 1600 Clifton Rd, Atlanta, GA 30333 USA; 2 PATH PAMO Plus, Lusaka, Zambia; 3 PATH Malaria Control and Elimination Partnership in Africa (MACEPA), Lusaka, Zambia; 4 PATH Malaria Control and Elimination Partnership in Africa (MACEPA), Seattle, WA USA; 5 University of California, San Francisco, CA USA; 6 U.S. President’s Malaria Initiative, U.S. Centers for Disease Control and Prevention, Lusaka, Zambia; 7 Malaria Branch, U.S. Centers for Disease Control and Prevention, Atlanta, GA USA; 8 Zambia Ministry of Health National Malaria Elimination Center, Lusaka, Zambia

**Keywords:** Malaria case management, Community health workers, *Plasmodiumfalciparum* prevalence, Healthcare seeking, Malaria

## Abstract

**Background:**

Malaria community case management (CCM) can improve timely access to healthcare, and CCM programmes in sub-Saharan Africa are expanding from serving children under 5 years (CU5) only to all ages. This report characterizes malaria case management in the setting of an age-expanded CCM programme in Chadiza District, Zambia.

**Methods:**

Thirty-three households in each of 73 eligible communities were randomly selected to participate in a household survey preceding a trial of proactive CCM (NCT04839900). All household members were asked about fever in the prior two weeks and received a malaria rapid diagnostic test (RDT); those reporting fever were asked about healthcare received. Weighted population estimates were calculated and mixed effects regression was used to assess factors associated with malaria care seeking.

**Results:**

Among 11,030 (98.6%) participants with RDT results (2,357 households), parasite prevalence was 19.1% by RDT; school-aged children (SAC, 5–14 years) had the highest prevalence (28.8%). Prior fever was reported by 12.4% of CU5, 7.5% of SAC, and 7.2% of individuals ≥ 15 years. Among those with prior fever, 34.0% of CU5, 56.0% of SAC, and 22.6% of individuals ≥ 15 years had a positive survey RDT and 73.7% of CU5, 66.5% of SAC, and 56.3% of individuals ≥ 15 years reported seeking treatment; 76.7% across all ages visited a CHW as part of care. Nearly 90% (87.8%) of people who visited a CHW reported a blood test compared with 73.5% seen only at a health facility and/or pharmacy (p < 0.001). Reported malaria treatment was similar by provider, and 85.9% of those with a reported positive malaria test reported getting malaria treatment; 66.9% of the subset with prior fever and a positive survey RDT reported malaria treatment. Age under 5 years, monthly or more frequent CHW home visits, and greater wealth were associated with increased odds of receiving healthcare.

**Conclusions:**

Chadiza District had high CHW coverage among individuals who sought care for fever. Further interventions are needed to increase the proportion of febrile individuals who receive healthcare. Strategies to decrease barriers to healthcare, such as CHW home visits, particularly targeting those of all ages in lower wealth strata, could maximize the benefits of CHW programmes.

**Supplementary Information:**

The online version contains supplementary material available at 10.1186/s12936-024-05047-1.

## Background

Malaria community case management (CCM), in which CHWs provide malaria health education, diagnostic testing with malaria rapid diagnostic tests (RDTs) for symptomatic individuals, and treatment for uncomplicated malaria with artemisinin-based combination treatment (ACT), has become a cornerstone of malaria control in sub-Saharan Africa. To further leverage CCM activities to reduce malaria related morbidity and mortality, some countries in sub-Saharan Africa are expanding CHWs’ roles from providing malaria care for under 5 year olds only to all ages, or age-expanded CCM [1, 2].

Gains in treatment seeking for fever among children under 5 years of age have largely plateaued in malaria endemic regions in sub-Saharan Africa, and gaps in appropriate malaria treatment continue to be identified for this age group [3–5]. Malaria care from CCM programmes can face challenges, including insufficient densities and availability of CHWs, difficulty in supervising and retaining trained CHWs, funding shortages, and RDT and ACT stock-outs [6]. However, there is substantial national and subnational heterogeneity in CCM programmes. Successful programmes have increased appropriate treatment-seeking for fever and malaria case management for children under 5 years of age, in some cases, exceeding appropriate malaria treatment rates at public health facilities [7]. In Zambia, malaria CCM scale up has been a national priority and associated reductions in severe malaria and mortality have been observed using routine surveillance data [8]. With diverse levels of CCM effectiveness observed for children under 5 years in sub-Saharan Africa, and as countries consider implementing age-expanded CCM for malaria, quantifying healthcare coverage and care quality across the age-spectrum will be needed to measure impact.

The objective of this report was to describe treatment-seeking for fever and malaria care quality in the setting of a well-resourced, age-expanded CCM program in Chadiza District, Eastern Province, Zambia. Data were used from a large household survey conducted prior to a cluster randomized trial of proactive community case management (ProCCM trial), where CHWs visit households weekly to identify suspected malaria cases and provide case management. This household survey was an opportune setting to characterize malaria burden among individuals of all ages and identify gaps in receipt of guidelines-based malaria treatment that may require additional targeted interventions.

## Methods

### Study setting and household selection

Chadiza District, Eastern Province, Zambia is a rural district bordering Mozambique with 111,069 residents on the most recent census [9]. It was served by 21 health facilities and 161 CHWs in 2021. It was chosen as the site of the ProCCM trial (NCT registration number: NCT04839900), a cluster-randomized controlled trial of proactive community case management, due to its moderate malaria burden (Eastern Province *Plasmodium falciparum* parasite prevalence by RDT in children under 5 years in 2021 was 24.8% [10]), and age-expanded CCM (established 2019) with 1 at least CHW per 500 community members. Vector control interventions included long-lasting insecticidal nets (LLINs) distributed through routine channels targeting pregnant women and children and by tri-annual mass campaigns, as well as annual indoor residual spraying (IRS) campaigns (see Table 1 for coverage).

**Table 1:** Household characteristics

Characteristic	Unadjusted	Population adjusted proportion or value (95% CI)
Demographics		
Number household heads	2,357	
Households per cluster, median (IQR)	33 (32–34)	
Members per cluster, median (IQR)	155 (140–169)	
Female head, *n* (%)	1332 (56.5%)	56.0% (53.3–58.8%)
Age of head in years, median (IQR)	38.6 (28.6–50.4)	38.6 (37.5–39.4)
Head education, *n* (%)		
None	716 (30.4%)	31.5% (28.4–34.5%)
Primary	1167 (49.5%)	49.9% (47.0–52.7%)
Secondary or higher	474 (20.1%)	18.7% (15.9–21.4%)
Members per household, median (IQR)	5 (IQR: 3–6)	4 (4–5)
Household construction		
Natural or rudimentary floor^1^	1598 (67.8%)	68.1% (63.3–72.9%)
Natural or rudimentary roof^2^	718 (30.5%)	31.9% (26.8–36.9%)
Unprotected or surface water source^3^	259 (11.0%)	9.4% (5.7–13.0%)
Latrine toilet	2183 (92.6%)	93.7% (90.3–97.1%)
Electricity available	143 (6.1%)	4.9% (2.1–7.8%)
Malaria control measures available		
At least 1 LLIN present in the household, *n* (%)	1362 (57.8%)	57.9 (53.3–62.4)
Percent with IRS within last year by cluster, *n* (%)	2099 (89.1%)	89.8% (87.6–92.0%)
Distance to healthcare provider		
Meters to nearest CHW, median (IQR)	548 (IQR: 248–1148)	530 (410–727)
Meters to nearest public health facility, median (IQR)	3291 (IQR: 1728–4353)	3352 (2915–3817)
Community health worker baseline knowledge and services		
Location of CHW known, *n* (%)	2,254 (95.6%)	96.2% (94.1–98.3%)
Reported frequency of CHW home visits^4^		
Weekly	146 (6.2%)	5.1% (3.2–7.1%)
Every 2 weeks	167 (7.1%)	6.7% (3.9–9.6%)
Monthly	569 (24.1%)	25.8% (19.1–32.5%)
Every 2–6 months	35 (1.5%)	1.9% (1.1–2.6%)
Every 6–12 months	55 (2.3%)	2.4% (1.2–3.7%)
Has not visited household	1256 (53.3%)	52.7% (45.6–59.8%)
Other	81 (3.4%)	3.2% (1.3–5.2%)
Don’t know	48 (2.0%)	2.1% (0.1–3.1%)
Reported services provided by CHW, *n* (%)		
Malaria testing^5^	2271 (96.4%)	97.0% (94.8–99.2%)
Malaria treatment^6^	2231 (94.7%)	95.3% (93.0–97.6%)
Antibiotic treatment^7^	148 (6.3%)	6.7% (4.1–9.3%)
Health education^8^	2029 (86.1%)	87.3% (83.2–91.5%)

^1^Natural/Rudimentary Floor Includes: Earth/Sand, Dung, Wood Planks, Palm/Bamboo, Clay. Finished includes: Parquet/polished wood, Vinyl or asphalt strips, ceramic tiles, cement, carpet, other

^2^Natural/Rudimentary Roof Includes: Thatch/Leaf, Sticks and Mud, Rustic Mat/Plastic Sheet, Reed/Bamboo, Would Planks, Wood, No roof and Thatch and iron. Finished includes: Iron sheets, Corrugated iron, Calamine/Cement Fiber, Cement/Concrete, Roofing Shingles

^3^Unprotected or surface water source: Unprotected well, Unprotected spring, Surface Water. Protected source: Piped into Dwelling, Piped into Yard/plot, Public Tap/Standpipe, Tube well or Borehole, Protected Well, Protected Spring

^4^46 households did not know the CHW visit frequency

^5^10 households missing malaria testing provided by CHW response

^6^55 households missing malaria treatment provided by CHW response

^7^304 households missing antibiotic treatment provided by CHW response

^8^87 households missing health education provided by CHW response

During the survey described in this report, CHWs provided malaria case management for symptomatic clients who requested care (either in the client’s home or outside the home). If a client had malaria symptoms, an RDT was done. ACTs were given if the RDT was positive. If the RDT was negative, the client would be referred to a health facility for further evaluation. Periodic CHW home visits could occur to deliver malaria related education. Periodic CHW home visits to screen and test symptomatic individuals (proactive case detection) were not in place.

### Sampling frame: cluster and household selection for household survey

**Fig. 1:**
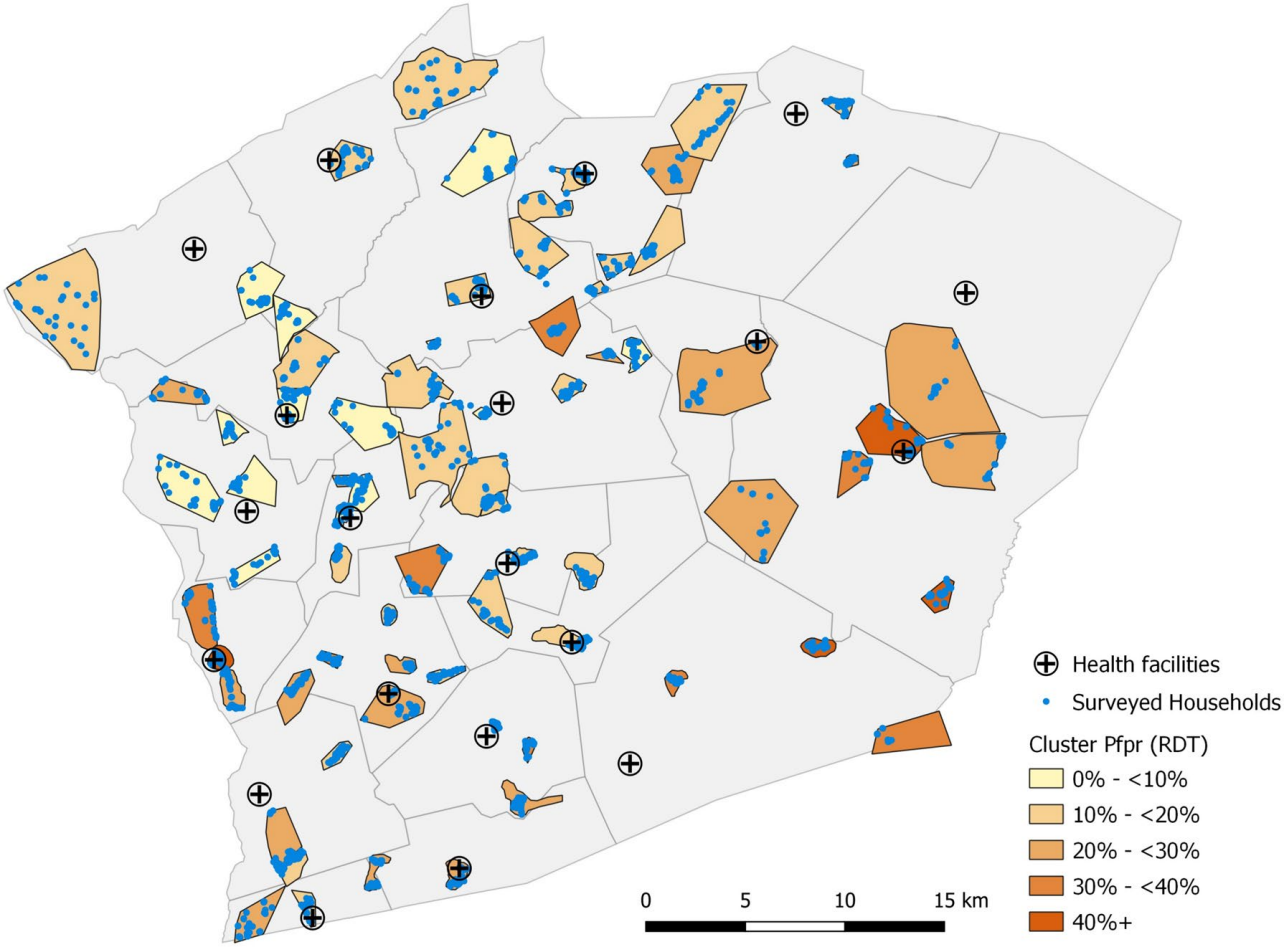
Locations and parasite prevalence of survey clusters, April—May, 2021. Clusters are outlined in black and households surveyed are blue points. Parasite prevalence by RDT indicated by shading of the cluster and health facilities in the district are noted by the cross marker

Catchment areas of at least 300 individuals served by 1 CHW were defined using the GPS location of each of the 161 CHWs, and the Euclidean distance to nearby IRS eligible structures with resident numbers that had previously been enumerated in the Akros mSpray mapping database [11]. Clusters which had at least 300 residents served by at least 1 CHW, the CHW cared for < 500 individuals, and, when possible, the CHW resided at least 1 km away from a neighboring CHW were selected for further evaluation. Cluster population size and boundaries were confirmed by field teams and CHWs. Among the 161 CHW catchment areas, 73 clusters met the selection criteria for the survey (Fig. 1). Forty households in each cluster were randomly selected, and households were approached consecutively until at least 33 consented to be enrolled in the survey.

### Study procedures

The survey was conducted between April 21, 2021 and May 26, 2021 (during the high transmission period). Among consenting households, geographic coordinates were collected and the head of household was interviewed using a modified Malaria Indicator Survey questionnaire [10] which collected data on household size and demographic information, typical treatment-seeking behaviour, head of household education, malaria control intervention availability (LLINs and IRS), whether a CHW visited the household and how frequently these visits occurred (never, every 2 weeks, monthly, every 2–6 months, every 6–12 months), and socioeconomic status (Supplemental Methods). In addition to the head of household survey, any household member with a history of fever in the prior 2 weeks (including day of survey) was asked additional questions including about days since fever onset, types of healthcare providers accessed (public or private hospital, clinic, mobile clinic, pharmacy, CHW, shop or traditional healer), and on the malaria care cascade (whether the febrile individual sought treatment, if a test was conducted, the result of the test, and medications received). All consenting household members received a *P. falciparum-*specific rapid diagnostic test (SD-Bioline™ Malaria Ag Pf). Individuals with a fever in the prior 2 weeks and a positive survey RDT were considered likely to have had malaria within the prior 2 weeks.

### Data analysis

Data were analysed using R version 4.2.3 (R Foundation for Statistical Computing, Vienna, Austria). Population estimates with 95% confidence intervals were calculated using the srvyr package (version 1.2.0) with a cluster design effect and survey weights. Chi-squared tests with the Rao-Scott correction were used to determine statistical significance for descriptive comparisons. Malaria care cascades for participants with reported fever in the prior 2 weeks were stratified by age group (age < 5, 5–14, ≥ 15 years) and survey RDT result. Mixed effects logistic regression, with a random intercept for cluster, was used to evaluate associations between participant characteristics and odds of a positive *P. falciparum* specific RDT (outcome 1) for the entire study population. Covariates for infection included age group, sex, wealth tertile as determined by a principal component analysis adapted from the Zambia Malaria Indicator Survey [10], household head education, household availability of LLINs or IRS, and reported frequency of CHW home visits. Among those who reported fever in the prior 2 weeks, mixed effects logistic regression was also used to evaluate covariate associations with whether or not treatment was accessed (treatment seeking, outcome 2). Covariates for treatment seeking included age group, sex, wealth tertile, household head education, survey RDT result, reported days since fever onset, reported frequency of CHW home visits, and Euclidean distance as calculated by geographic coordinates of the household to the closest public healthcare source (CHW residence or public health facility). The lme4 package (version 1.1–34) was used for all models.

## Results

### Survey and participant demographics

In total, 2,915 (25%) eligible household structures were randomly selected, and 2,458 (84.3%) were visited at least once. Among households visited, 2,357 (95.9%) households consented to participate in the survey. A median of 33 households (IQR: 32–34; range: 11–39) and 155 individuals (IQR: 140–169; range: 50–236) were surveyed per cluster. Data were available from 11,185 (97.4%) of the 11,486 reported household residents (Table 1; Supplemental Fig. 1); RDT results were available from 11,036 (98.6%) individuals.

### Parasite prevalence

**Fig. 2:**
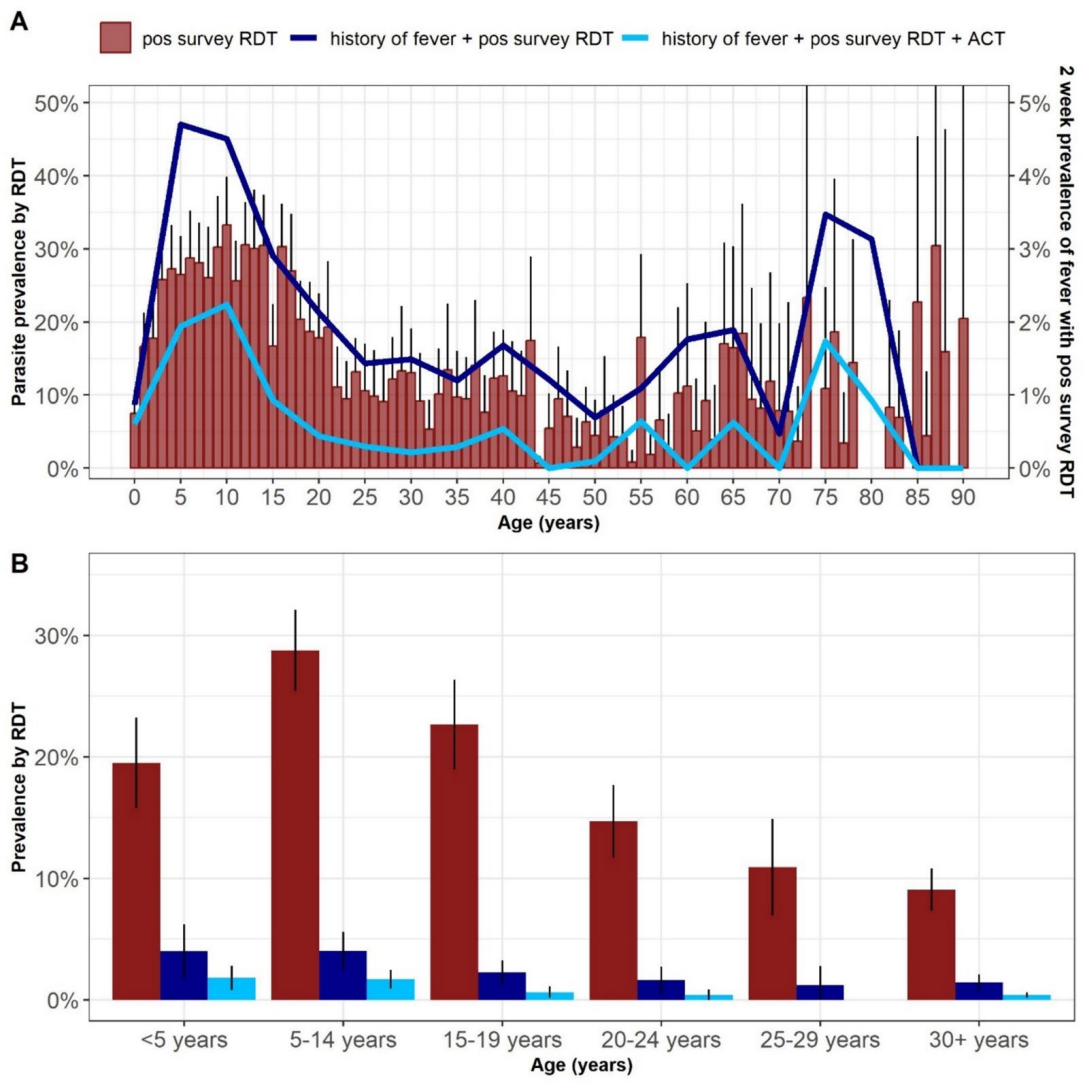
**A** Parasite prevalence and 2-week prevalence of fever with positive survey RDT. The red bars represent the population adjusted parasite prevalence by age with the vertical lines representing the upper bound of the 95% confidence interval and the percentage shown on the left axis. The dark blue line is the 2-week prevalence of fever with positive survey RDT by age group (right axis). The light blue line is 2-week prevalence of fever with positive survey RDT plus reported receipt of artemisinin-based combination therapy (ACT) in the prior 2 weeks (right axis). **B** Proportion of household members with a positive study RDT (red), 2-week prevalence of fever with a positive survey RDT (dark blue) and 2-week prevalence of fever with positive survey RDT and reported receipt of an ACT (light blue). The bars indicate population adjusted prevalence by age group and the black lines indicate the 95% confidence interval

*Plasmodium falciparum* prevalence by RDT was 19.1% (95% CI 16.6–21.7%) across all age groups. Parasite prevalence varied across clusters (median: 18.3%, IQR: 11.1–25.0%, range: 3.4%-55.7%, Fig. 1) and by age (Fig. 2). School aged children (SAC, 5–14 years of age) had the highest parasite prevalence (28.8% [25.4–32.1%]), compared with those under 5 years (19.5% [15.8–23.2%]) and ≥ 15 years (12.9% [10.8–15.0%]; Table 2). After accounting for age group, gender, household wealth tertile, and head of household education level (Supplemental Table 1), household ownership of at least one LLIN was associated with a lower odds (OR: 0.73 [0.70–0.75]) of an individual having a positive RDT. Reporting IRS within the last year was not associated with RDT positivity. Compared with never receiving home visits, reported receipt of home visits from a CHW was associated with lower odds of parasitaemia if they occurred every 2–6-months (OR: 0.72 [0.66–0.79]), while monthly or more frequent CHW home visits were not associated with odds of parasitaemia (OR: 1.00 [0.95–1.03]).

**Table 2:** Study participant demographics, survey RDT results, and care seeking practices by age group among individuals with reported fever in the prior 2 weeks^1^

Characteristic	Age in years (n, %)		
	< 5 (n = 2141, 19.1%)	5- < 15 (n = 3483, 31.1%)	> 15 (n = 5561, 49.7%)
Female, weighted % (95% CI)	52.4 (49.5–55.4)	56.1 (53.6–58.5)	62.0 (60.7–63.4)
Study RDT conducted^2^, *n* (%)	2108 (98.4%)	3434 (98.6%)	5497 (98.8%)
Study RDT positive^3^, *n* (%)	441 (20.9%)	996 (29.0%)	728 (13.2%)
Population adjusted parasite prevalence	19.5% (15.8–23.2%)	28.8% (25.4–32.1%)	12.9% (10.8–15.0%)
Fever in the prior 2 weeks^4^, *n* (%)	281 (13.2%)	268 (7.8%)	405 (7.3%)
Population adjusted fever in the prior 2 weeks	12.4% (8.3–16.7%)	7.5% (4.9–10.0%)	7.2% (5.2–9.2%)
Malaria diagnostics and treatment among participants with fever in the prior 2 weeks			
Sought care in the prior 2 weeks^5^	73.7% (65.8–81.7%)	66.5% (59.4–73.7%)	56.3% (44.7–67.9%)
Of those who sought care, care included CHW	72.1% (61.7–82.6%)	76.6% (64.8–88.5%)	80.9% (72.9–88.8%)
Of those who sought care, reported receiving a malaria test^6^	84.3% (77.9–90.7%)	91.6% (85.5–97.7%)	84.4% (78.4–90.4%)
Of those who sought care with CHW, reported receiving a malaria test	90.6% (85.6–95.7%)	92.1% (84.7–99.5%)	88.8% (83.8–93.8%)
Reported positive malaria test among all with prior fever	37.4% (29.8–44.9%)	47.8% (39.1–56.6%)	23.3% (16.6–30.1%)
Positive survey RDT among all with prior fever	34.3% (22.0–46.5%)	57.6% (50.3–64.8%)	25.0% (17.8–32.1%)
Reported receiving an ACT among all with fever and positive survey RDT	47.7% (33.9–61.5%)	45.2% (35.3–55.0%)	25.1% (13.9–36.3%)
Reported receiving an ACT among all with fever and negative survey RDT	29.0% (23.0–34.9%)	42.7% (28.2–57.2%)	19.8% (13.0–26.6%)

^1^unless otherwise noted, all percentages are population adjusted percentages (95% confidence interval)

^2^146 participants did not receive an RDT

^3^RDT results not available for 155 participants

^4^74 missing information on history of fever in the prior 2 weeks

^5^97 individuals with fever missing information on malaria-care cascade

^6^15 individuals with missing testing data on malaria testing

### Fever with *Plasmodium* falciparum infection by survey malaria RDT

The overall prevalence of reported fever in the prior 2 weeks was 8.3% (5.8–10.8%). Children under 5 years of age were the most likely to report a fever compared to either SAC (p < 0.001) or individuals ≥ 15 years of age (p < 0.001, Table 2). In total, 954 individuals reported fever in the prior 2 weeks and 857 (89.8%) answered questions about treatment seeking. Among individuals with fever and treatment seeking data, the majority, 87.2% (81.2–93.2%), reported fever onset between 0 and 7 days before the study visit.

Although nearly a fifth of the population was parasitaemic by RDT at the time of the survey, only 14.8% (9.6–20.0%) of the RDT positive population reported fever in the prior two weeks (suggesting a current or recent clinical malaria infection); and only 5.6% (3.5–7.7%) of all survey RDT positive individuals reported receiving an ACT in the prior 2 weeks. Among survey RDT negative individuals, 6.4% (4.6–8.3%) reported a prior fever and 4.1% (2.8–5.4%) had received an ACT. Over half of SAC with fever in the prior two weeks had a positive survey RDT (57.6% [50.3–64.8], Table 2).

### Treatment-seeking for fever

**Fig. 3:**
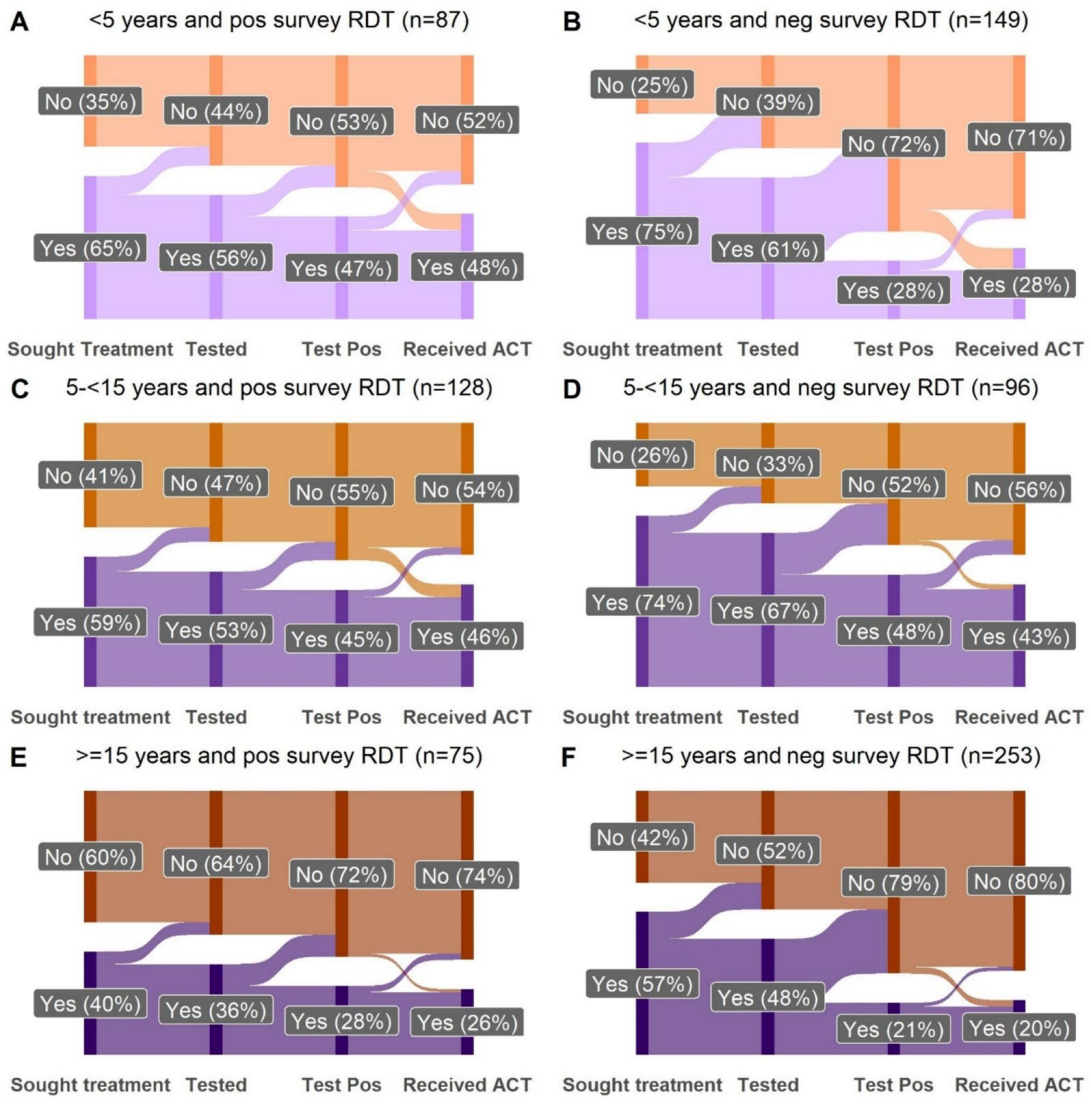
Malaria-care cascades for study participants who reported fever in the prior two weeks, stratified by age group and survey RDT result (**A**–**F**). The number of study participants contributing to each diagram are listed in the titles. All percentages are the median population adjusted percentages. Responses of “not known” were excluded in the graphic as they make up less than 2% of the responses. Sought treatment indicates a report of receiving healthcare in the formal sector, though it could have been delivered at the home by CHWs or outside the home. *Pos* Positive, *neg* negative

A total of 807 individuals (84.6%) responded to treatment seeking questions and received a survey RDT. Overall, 64.4% (56.8–71.9%) of household members who reported fever in the prior 2 weeks reported accessing healthcare. Individuals under 5 years of age (Fig. 3), those in households in the highest wealth tertile, longer duration from fever onset to the study visit, and reporting monthly or more frequent CHW home visits at the household level were associated with an increased odds of treatment seeking for fever (Table 3). There were trends towards lower odds of treatment seeking among individuals with a positive survey RDT as compared with a negative RDT (OR: 0.68 [0.46–1.00]) and among those who lived more than 3000 m (or approximately 30 min walking distance) from the nearest CHW or health facility (OR 0.27 [0.06–1.35]).

**Table 3:** Factors associated with healthcare-seeking among individuals with fever

Characteristic	N	Proportion formal healthcare seeking^1^ (95% CI)	Odds of seeking care			
			Unadjusted odds ratio (95% CI)	p-value	Adjusted odds ratio (95% CI)	p-value
Sex						
Male	329	58.3 (47.7–68.8)	–	–	–	–
Female	478	63.8 (57.3–70.4)	1.23 (0.89–1.69)	0.21	–	–
Age group						
< 5 years of age	241	70.4 (61.9–78.9)	–	–	–	–
5–14 years of age	226	64.7 (57.0–72.5)	0.61 (0.40–0.95)	0.03	0.53 (0.33–0.86)	0.01
≥ 15 years of age	340	53.3 (42.0–64.7)	0.43 (0.29–0.64)	< 0.001	0.34 (0.22–0.54)	< 0.001
Wealth tertile						
Lowest	262	54.2 (42.7–65.7)	–	–	–	
Middle	250	62.5 (52.1–72.8)	1.25 (0.84–1.87)	0.16	1.29 (0.83–2.01)	0.26
Highest	295	67.6 (60.1–75.0)	1.91 (1.28–2.84)	0.002	1.90 (1.21–2.98)	0.005
Household head education						
None	290	55.7 (42.3–69.1)	–	–	–	–
Primary	370	63.9 (57.5–70.2)	1.15 (0.80–1.66)	0.45	–	–
Secondary or more	147	66.1 (53.6–78.6)	1.51 (0.92–2.47)	0.10	–	–
Study RDT result						
Negative	511	65.6 (58.9–72.3)	–	–	–	–
Positive	296	54.5 (45.4–63.7)	0.64 (0.46–0.91)	0.01	0.68 (0.46–1.00)	0.05
Days since fever onset						
0–1 days	117	41.7 (27.2–56.1)	–	–	–	–
2–3 days	322	56.8 (45.6–67.9)	3.06 (1.76–5.30)	< 0.001	3.57 (1.95–6.53)	< 0.001
4–6 days^3^	133	62.0 (49.2–74.7)	4.58 (2.40–8.74)	< 0.001	5.44 (2.66–11.1)	< 0.001
7–14 days	240	78.1 (71.7–84.5)	8.37 (4.58–15.28)	< 0.001	9.32 (4.86–17.9)	< 0.001
Frequency of CHW visits to household^4^						
Every two months or less often	563	60.9 (50.5–71.3)	–	–	–	–
Monthly or more often	224	63.4 (55.1–71.7)	1.54 (1.03–2.30)	0.03	1.63 (1.06–2.53)	0.03
Don’t Know	20	57.5 (49.3–65.6)	–	–	–	–
Distance from nearest CHW or public health facility^2^						
≤ 3,000 m	750	62.0 (54.0–70.2)	–	–	–	–
> 3,000 m	51	49.0 (41.9–56.0)	0.42 (0.11–1.51)	0.08	0.27 (0.06–1.25)	0.10

^1^Population adjusted percentage (95% confidence interval) among those with fever, study RDT results, and completed questions on care seeking

^2^Euclidean distance from community health worker or health facility, whichever is closer, with 3000 m requiring approximately 30 min of walking time, 6 individuals missing the distance to the nearest facility

^3^Unknown date of onset recoded as median value (4 days)

^4^A response of “Other” considered in the reference group, 20 individuals with response of “Don’t Know” excluded from the analysis

### Malaria case management cascade

**Fig. 4:**
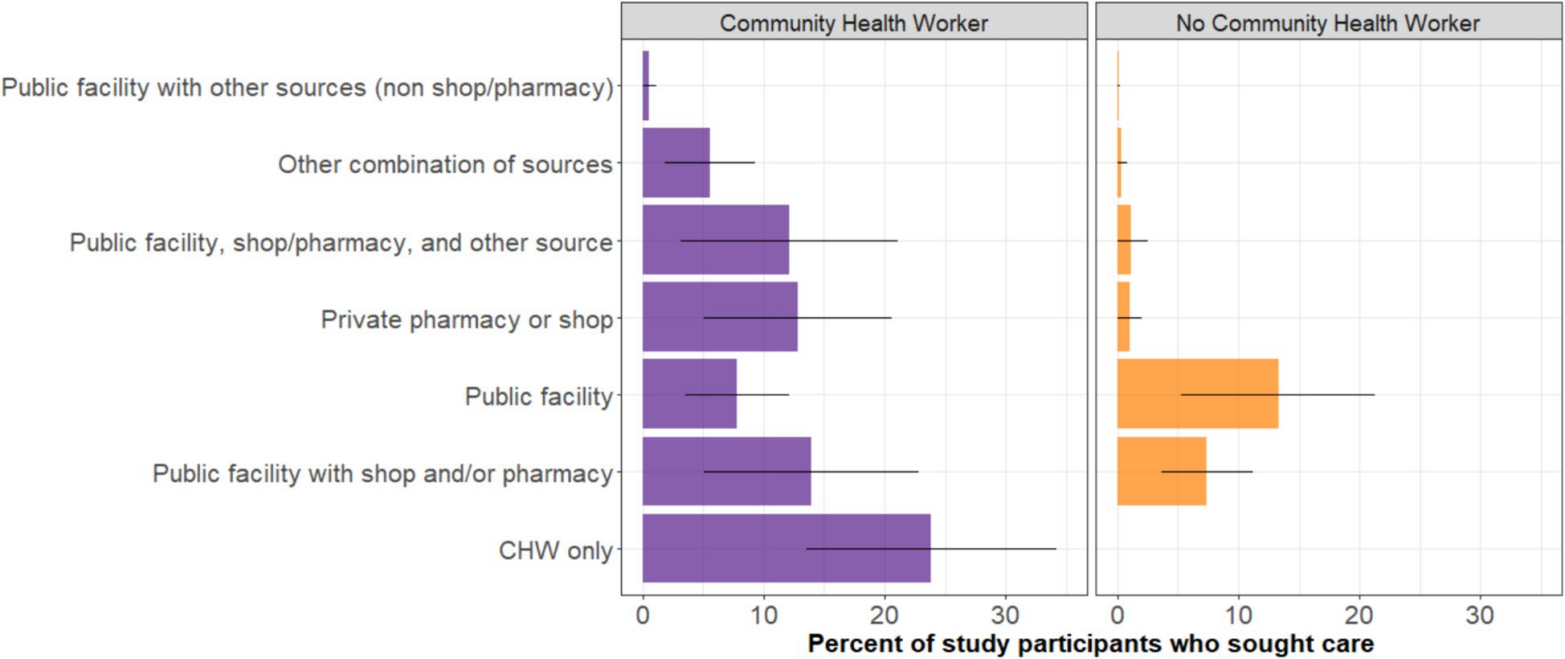
Reported formal healthcare sources accessed for febrile illness among those who sought healthcare, stratified by whether a community health worker (CHW) was reported as accessed. The bars indicate the population adjusted median percentage, the black lines encompass the 95% confidence interval of the estimate. In the purple bars, a CHW was accessed in addition to the other providers listed. In the yellow bars, a CHW was not reported as accessed

Among those who sought treatment for fever (n = 559), 76.7% (68.4–84.9%) received care from a CHW, either exclusively (23.9% [13.5–34.2%]) or in combination with other sources (52.8% [39.5–66.1]) (Fig. 4). Time between fever onset and treatment seeking was similar between those who accessed a CHW as part of care and those who used non-CHW-based care (median: 2 days, IQR 2–3 and 2, IQR 2–3). Utilization of CHWs was similar regardless of survey RDT result (p = 0.18) or age group (p = 0.19, Table 2). When healthcare services were required in addition to a CHW, a public health facility (health post, health center, or hospital) plus a private pharmacy or shop (13.9% [5.1%–22.8%]) were most commonly accessed, followed by a CHW plus a private pharmacy or shop only (12.8 [5.1%–20.6%]) (Fig. 4). Non-CHW-based care usually included a government health facility alone (13.3% [5.3%–21.3%]) or in combination with a shop and/or pharmacy (7.4% [3.7–11.2%]). A higher proportion of participants sought multiple healthcare sources if a CHW was accessed (68.9% [54.9–82.8%]) than if a CHW was not accessed (37.1% [17.6–56.5%], p = 0.002). Similar proportions of participants sought multiple sources of care if a CHW was accessed regardless of the survey RDT result (positive RDT: 58.9% [44.0–73.8%], negative RDT: 65.7% [48.6–82.9%], p = 0.27).

**Fig. 5:**
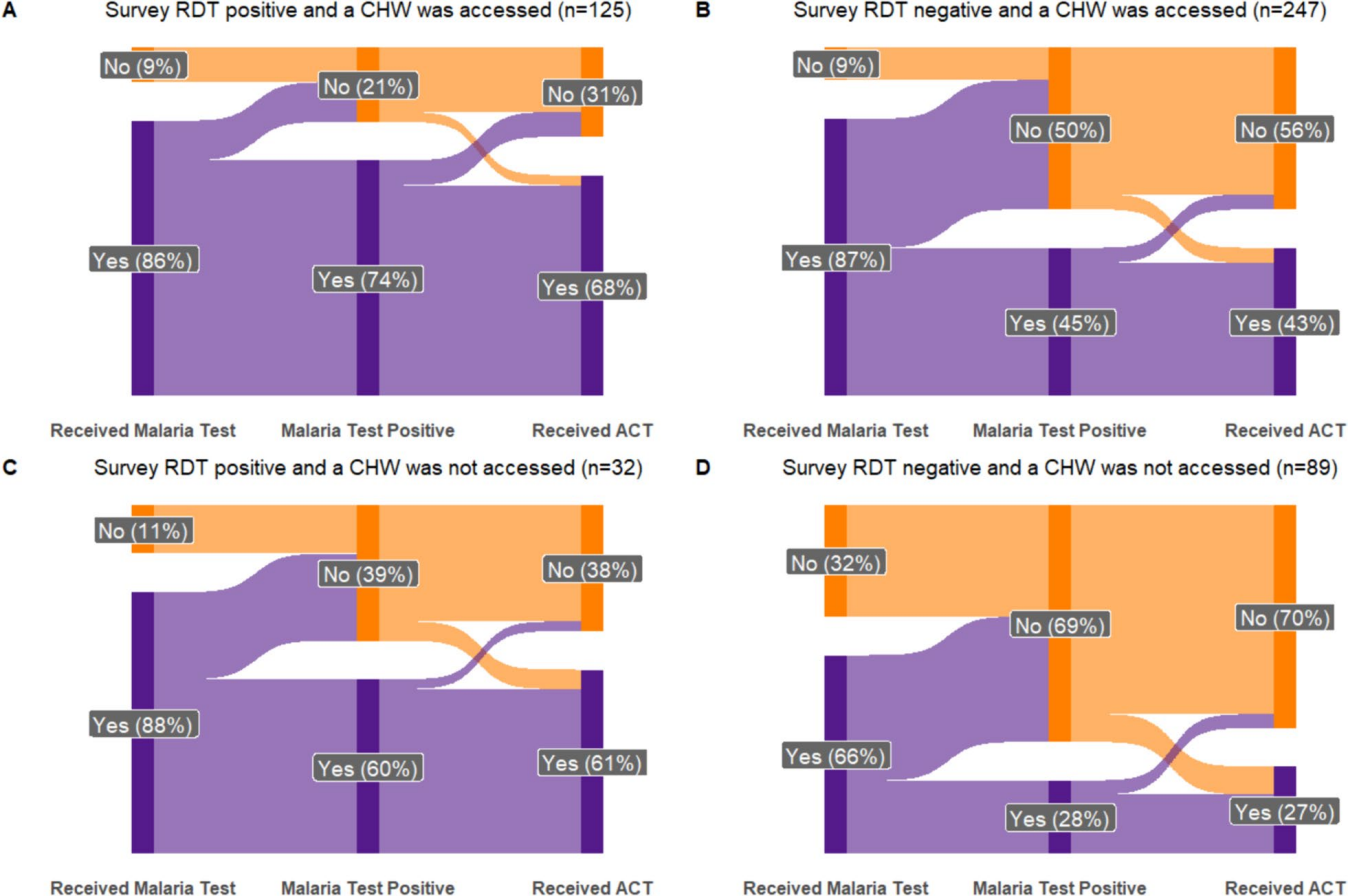
Malaria-care cascade for study participants who reported fever in the prior 2 weeks and sought care, stratified by survey RDT result and whether a CHW was accessed as part of the healthcare received (**A**–**D**). The number of study participants contributing to each diagram are listed in the titles. All percentages are the median population adjusted percentage. Percentages do not add up to 100% as survey responses of ‘not known’ were excluded from the flow diagram for clarity but included for calculation of the adjusted percentages

Those who reported accessing a CHW as part of their care were more likely to report receiving a malaria test (86.7% [82.2–91.3%]) compared with those who did not use a CHW (73.5% [63.9–83.1], p = 0.003). However, among individuals with a positive survey RDT (who were more likely to have had malaria as the cause of fever) the proportion tested was similar between those who visited CHW and those that did not (Fig. 5). Among individuals who reported a positive malaria test, similar proportions received an ACT if a CHW was part of malaria care or not (86.5% [79.5–93.5%] vs 83.1% [63.5–100%], p = 0.72).

Among all participants with prior fever and a positive survey RDT, 40.1% (95% CI 32.0–48.1%) reported having received an ACT, with higher receipt of ACT in the younger age groups (< 5 years: 47.7% [33.9–61.5%]; 5–14 years; 45.2% [35.3–55.0%]; > 15 years: 25.1% [13.9–36.3%], p = 0.01) (Fig. 3). Among participants with fever and a negative survey RDT, suggesting a non-malaria diagnosis for fever, 26.9% (21.1–32.8%) reported having received an ACT, with the highest reported treatment rates in the SAC group (Table 2).

## Discussion

In this novel large representative survey where all household members had been eligible for CCM prior to the survey and all received an RDT during the survey, there was a substantial burden of parasitaemia across the age spectrum, but the vast majority of those with a positive survey RDT did not report having a fever in the prior 2 weeks. Depending on the age group, between half and two thirds of individuals with prior fever and a positive survey RDT sought treatment. Among individuals who sought treatment, the majority reported receiving guidelines-based malaria diagnostic testing and treatment, and three quarters of all individuals included a CHW as part of their care. Individuals 5 years of age and older, and those in the lowest wealth tertile had lower odds of seeking healthcare for fever.

Risk factors for parasitaemia in Chadiza District were similar to other moderate transmission settings in sub-Saharan Africa, and school aged children had the highest *P. falciparum* prevalence by RDT [12, 13]. However, compared to other malaria endemic settings, reported treatment seeking for children under 5 years of age was high, suggesting largely successful implementation of CCM for the highest risk group for severe malaria and mortality [14]. The survey identified additional malaria care delivery strengths, including that health care for fever was largely provided by the public sector, CHWs cared for over three quarters of treatment seekers for fever, and distance to a healthcare provider was not strongly associated with care-seeking.

However, some remaining gaps in healthcare access were identified. Treatment-seeking for fever among individuals 15 years of age and older (50% of the study population) and those in the lower wealth tertiles had the largest gap in coverage for malaria care [15]. Individuals over 15 years of age accounted for a third of all positive survey RDT results, but were half as likely to seek treatment for fever, and only a quarter of those with evidence of a recent malaria episode (recent fever + positive survey RDT) had received an ACT. Those in the highest wealth tertile had nearly twice the odds of treatment-seeking for fever compared to the lowest wealth tertile. Treatment-seeking is a complex process, with a country’s healthcare expenditure rates, overall access to routine healthcare (e.g. immunizations, prenatal care), education, the number, severity and duration of malaria symptoms, individual costs (both costs of care and opportunity costs), knowledge about malaria, and interpersonal support all associated with treatment-seeking [14, 16, 17]. Additional data which characterizes remaining barriers to treatment seeking for adults and the most socioeconomically vulnerable in a well-scaled CCM programme will be needed.

Proactive CCM, in which CHWs conduct active malaria case detection by screening for malaria symptoms including fever at regularly scheduled home visits (e.g. weekly) has been proposed as one strategy to increase coverage of appropriate malaria care across the age spectrum and decrease community level malaria transmission [18–20]. Although proactive malaria CCM was not in place in Chadiza District during the survey, if the household head reported monthly or more frequent home visits by a CHW, there were higher odds of treatment-seeking for fever. Household visits by CHWs in Chadiza District at this time could have occurred as part of passive detection of malaria (a participant requesting care for fever) or through periodic malaria education sessions, and the data available could not distinguish between these activities (Supplemental Methods). Regardless, an active case detection strategy would likely reach more individuals with malaria testing and treatment across the age and wealth spectrum. However, as only a minority of parasitaemic individuals of any age reported fever in the prior 2 weeks (15%) it is uncertain if proactive CCM would decrease malaria transmission in this setting.

Importantly, the majority of individuals who accessed a CHW also accessed 1–2 other healthcare sources (e.g. a public health facility, private pharmacy, and/or an informal shop). Identifying ways to reduce the number of healthcare visits needed to care for febrile individuals could reduce costs to the healthcare system and opportunity costs for healthcare seekers. The Malaria Indicator Survey-based survey tool did not differentiate between services provided by each healthcare provider that contributed to fever or malaria case management or delineate why multiple providers were accessed (e.g. accessibility, quality of care, stock-outs, type and severity of illness). Collecting population representative and qualitative data on which services are accessed by different community members and why multiple providers are used during care seeking for fever could help CCM programmes further optimize their service delivery to reduce barriers and costs associated with healthcare-seeking for fever [4].

Some individuals with a prior fever and a negative survey RDT, suggesting a non-malaria etiology for their fever, reported receiving a positive malaria test and an ACT. Although this finding is consistent with other surveys [4], it is unknown if these participants had a false positive malaria diagnostic test (microscopy or RDT), or if there was overreporting of positive malaria tests by study participants [21]; a reversion of the RDT to negative less than 2 weeks following treatment would likely account for only a minority of the discordance [22]. Overdiagnosis of malaria could delay treatment for alternative aetiologies and increase malaria case management program costs. Understanding the underlying causes of these discrepancies, particularly assessing whether they are related to participant recall, suboptimal healthcare practices or diagnostic test characteristics, could improve CCM programme effectiveness [6].

Using a pre-intervention household survey to assess CCM coverage and treatment-seeking was a strength of this study, as it allowed for description of treatment seeking for fever for 25% of households within clusters receiving age-expanded CCM. This study was powered to provide granular data, including data on treatment seeking and parasite prevalence, on individuals of all ages who are known to receive the intervention of interest. In order to assess CCM utilization, coverage effectiveness, identify resource needs, and readiness for age-expansion, targeted rich sampling in subnational regions may be needed for programmatic assessment. For such targeted surveys, revising the data collection instruments further to include data on services received at different providers, monetary and opportunity costs of seeking care, and reasons for using specific providers could assist in making survey results programmatically actionable.

This study was subject to several limitations. First, we used self-reported fever within the prior 2 weeks with a positive survey RDT as a marker for recent clinical malaria. Although some individuals may be misclassified by this definition, this is a group where anti-malarial treatment is indicated. Second, when multiple providers were accessed, the survey did not include questions on which providers administered malaria diagnostic testing and treatment. The high testing and treatment rates among those who accessed a CHW cannot be fully attributed to care from CHWs directly. Third, it is possible that some individuals with malaria symptoms but without a fever received an ACT, but this group was not questioned about healthcare-seeking as part of the survey. Finally, these data reflect the treatment-seeking behaviors and malaria burden in Chadiza District, Zambia, and may not be representative of other malaria endemic areas. Documenting the potential impacts of investment in and age-expansion of CCM may have applications in other moderate to high malaria transmission settings.

In this region with high CCM coverage serving symptomatic individuals of all ages, there was a high burden of parasitaemia across the age spectrum. Treatment-seeking for fever was high, but gaps were identified, particularly for individuals 5 years of age and older and of lower wealth. When care was accessed, CHW-based care led to high levels of malaria diagnostic testing and test-result based treatment with artemisinin-based combinations. However, refining our standard survey tools and sampling strategies to quantify reasons for seeking treatment from multiple different providers, barriers and facilitators for treatment-seeking, and more details on types of services provided could make survey data more actionable for highly utilized CCM programmes, such as in Chadiza District.

### Supplementary Information

Additional file 1.

Additional file 2.

## Data Availability

The datasets used and/or analyzed during the current study are available from the corresponding author on reasonable request.
