# Supplementary material for: Malaria community case management usage and quality of malaria care in a moderate Plasmodium falciparum burden region of Chadiza District, Zambia
Source: Malar J. 2024 Aug 1;23:226. doi: 10.1186/s12936-024-05047-1 (PMC11292954; doi:10.1186/s12936-024-05047-1)
Supplement: Supplementary file 1 — Additional file 1. [file 12936_2024_5047_MOESM1_ESM.docx]

**
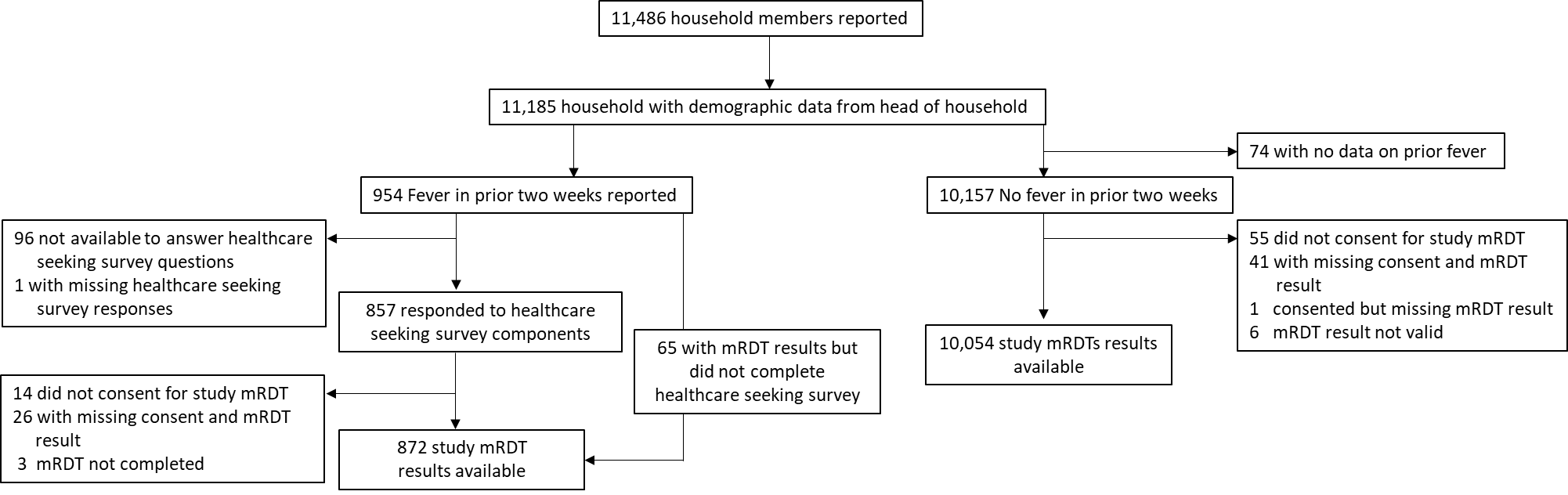
**

**Supplemental figure 1.** Study diagram.

**Supplemental table 1.** Risk factors for *P. falciparum* parasitemia as detected by mRDT.

| Characteristic | N | Parasite prevalence (95% CI) | Odds of parasitemia | | | |
| --- | --- | --- | --- | --- | --- | --- |
|  |  |  | Unadjusted odds ratio (95% CI) | p-value | Adjusted odds ratio (95% CI)* | p-value |
| **Age group & sex** | | | | | | |
| <5 years |  |  | - | - | - | - |
| Male | 1005 | 19.7 (15.6-23.7) | - | - | - | - |
| Female | 1102 | 19.4 (15.1-23.6) | 1.01 (0.94-1.08) | 0.86 | 1.02 (0.95-1.09 | 0.56 |
| 5-14 years |  |  | 2.39 (2.24-2.54) | <0.001 | 2.46 (2.31-2.63) | <0.001 |
| Male | 1505 | 32.2 (27.9-36.4) | - | - | - | - |
| Female | 1927 | 26.1 (22.5-29.7) | 0.67 (0.61-0.73) | <0.001 | 0.66 (0.62-0.70) | <0.001 |
| ≥15 years |  |  | 0.83 (0.78-0.89) | <0.001 | 0.86 (0.80-0.91) | <0.001 |
| Male | 2088 | 15.9 (13.1-18.7) | - | - | - | - |
| Female | 3403 | 11.0 (9.0-13.1) | 0.63 (0.58-0.69) | <0.001 | 0.61 (0.57-0.65) | <0.001 |
| **Wealth tertile** | | | | | | |
| Lowest | 3050 | 23.2 (19.5-26.9) | - | - | - | - |
| Middle | 3640 | 18.8 (16.1-21.3) | 0.91 (0.88-0.95) | <0.001 | 0.91 (0.88-0.95) | <0.001 |
| Highest | 4340 | 16.4 (13.7-19.3) | 0.80 (0.77-0.84) | <0.001 | 0.82 (0.79-0.86) | <0.001 |
| **Household head education** | | | | | | |
| None | 3528 | 23.1 (19.1-27.1) | - | - | - | - |
| Primary | 5364 | 17.9 (15.6-20.2) | 0.79 (0.76-0.82) | <0.001 | 0.83 (0.80-0.86) | <0.001 |
| Secondary or more | 2138 | 14.9 (12.4-17.4) | 0.66 (0.63-0.69) | <0.001 | 0.77 (0.73-0.82) | <0.001 |
| **LLINs** | | | | | | |
| None | 4526 | 22.9 (19.5-26.3) | - | - | - | - |
| ≥1 | 6504 | 16.4 (14.1-18.7) | 0.69 (0.67-0.72) | <0.001 | 0.73 (0.70-0.75) | <0.001 |
| **IRS** | | | | | | |
| None | 1093 | 19.3 (16.7-21.9) | - | - | - | - |
| Sprayed within 1 year | 9913 | 17.1 (12.1-22.0) | 0.94 (1.01-1.13) | 0.03 | 0.99 (0.94-1.06) | 0.87 |
| Unknown status | 24 | 13.5 (0-30.1) | - | - | - | - |
| **Reported frequency of CHW visits to household** | | | | | | |
| Never visited | 5878 | 20.3 (17.7-23.9) |  |  | - |  |
| Visits occur at 2-6 month intervals | 465 | 17.1 (12.8-21.3) | 0.72 (00.67-0.79) | <0.001 | 0.72 (0.66-0.79) | <0.001 |
| Visits occur monthly or more often | 4124 | 18.0 (15.3-20.6) | 0.95 (0.92-0.99) | 0.02 | 1.00 (0.91-1.09) | 0.86 |
| Other visit frequency | 370 | 9.3 (5.4-13.2) | 0.57 (0.50-0.64) | <0.001 | 0.57 (0.48-0.68) | <0.001 |
| Unknown visit frequency | 193 | 16.1 (3.3-28.9) | - | - | - | - |

*When CHW home visit frequency or spraying of IRS was unknown, these were excluded form the multivariate analysis (n=213 [1.9%])
